# The Effects of the COVID-19 Pandemic on Trauma Presentations in a Level One Trauma Center

**DOI:** 10.1177/0003134820973715

**Published:** 2021-05

**Authors:** Aditya K. Devarakonda, Chase J. Wehrle, Fairouz L. Chibane, Peter D. Drevets, Elizabeth D. Fox, Andrew G. Lawson

**Affiliations:** 2 1421 Medical College of Georgia at Augusta University, Augusta, GA, USA; 2 Department of Surgery, 1421 Medical College of Georgia, Augusta, GA, USA

## Abstract

**Background:**

Over 28 million confirmed cases of COVID-19 have been reported to date, resulting in over 900 000 deaths. With an increase in awareness regarding the virus, the behavior of general population has changed dramatically. As activities such as driving and hospital presentation patterns have changed, our study aimed to assess the differences in trauma case variables before and during the COVID-19 pandemic.

**Methods:**

Trauma data for the period of March 1st-June 15th were compared for the years 2015-2019 (pre-COVID) and 2020 (COVID). The data were analyzed across the following categories: injury severity score, injury mechanism, motor vehicle crashes (MVCs) vs. other blunt injuries, alcohol involvement, and length of hospital stay.

**Results:**

The median injury severity score pre-COVID and during COVID was 9, representing no change. There was no difference in overall distribution of mechanism of injury; however, there was a significant decrease in the percentage of MVCs pre-COVID (36.39%) vs. COVID (29.6%, *P* < .05). Alcohol was significantly more likely to be involved in trauma during COVID-19 (*P* < .05). The mean hospital stay increased from 3.87-5.4 days during COVID-19 (*P* < .05).

**Discussion:**

We saw similar results to prior studies in terms of there being no change in trauma severity. Our observation that motor vehicle collisions have decreased is consistent with current data showing decreased use of motor vehicles during the pandemic. We also observed an increase in alcohol-related cases which are consistent with the reported changes in alcohol consumption since the pandemic began.

## Introduction

There has not been a pandemic to the scale and impact of the COVID-19 in recent history. As of September 13, 2020, the World Health Organization reported over 28 million confirmed cases and 900 000 deaths worldwide.^1^ The Centers for Disease Control reports over 6.4 million cases and 193 000 deaths.^2^ Although many mitigating measures, such as masks and social distancing, have been implemented to curb the transmission, the virus has already spread extensively throughout the world.^3^ The public has become acutely aware of the infectious processes by which the disease is transmitted, and as such, general behaviors of the population have changed.^4^ Also, there has been a significant change in the organizational structure and practices of hospitals across the United States.^5^

With the overall change in public behavior, the injury patterns seen in acute care settings such as emergency departments are likely subject to change. Motor vehicle use has declined significantly through the COVID-19 pandemic.^6^ As social distancing guidelines promote individuals staying home, the frequency of various injury mechanisms may shift.

It is well documented that social isolation increases the risk for substance abuse, specifically alcoholism.^7^ COVID-19 has been shown to correlate with an increase in alcohol sales as well as an increase in risk for relapse in alcoholics.^8,9^ Individuals have also recognized hospitals as a major potential source of COVID-19 and as such, certain hospital services such as emergency department traffic have seen significantly decreased volume during the pandemic.^10^ The increase in alcohol and substance abuse may be a result of the increased mental health strain on the population as a whole. Depression symptoms during the pandemic have been shown to be about 3 times greater than prior to the pandemic.^11^

This study investigates the impact of COVID-19 on the changes in various factors of trauma cases such as the mechanisms and severity of injury, the frequency of the involvement of alcohol, and the length of stay after admission. Our aim is to identify which factors have changed before and after the pandemic. This may allow us to provide trauma centers with a better idea of the types of changes that may occur during future pandemics so they may adjust accordingly.

## Methods

All study protocols were approved by the institutional review board at Augusta University. A retrospective study was conducted using institutional trauma data from an academic level 1 trauma center. The pre-COVID-19 data were collected from trauma activations during the period from March 1st-June 15th from 2015-2019. The COVID-19 period was considered the time between March 1st and June 15th in 2020. Several variables were examined between the 2 periods including the median injury severity score (ISS), the distribution of the injury mechanism, the distribution of motor vehicle crashes (MVCs) compared to other blunt injuries, frequency of alcohol involvement, and the hospital length of stay. There were 4 mechanisms of injuries which were compared: blunt trauma, burns, penetrating injuries, and other traumas.

The ISS and hospital length of stay are nonparametric variables and as such were compared via a Mann-Whitney U test or a Wilcoxon rank sum test to determine significance. For the distributions of injury mechanisms, MVC vs. other blunt injuries, and alcohol involvement, a chi-squared test was used to determine significance. All statistical analyses were conducted in R version 4.0.0 from R Studio R based in Boston, Massachusetts.

## Results

There were 3033 trauma activations during the pre-COVID-19 time period and 574 activations during the COVID-19 period. Data were collected from the same hospital over the same time period in different years before and after the pandemic.

First, the differences between pre-COVID and COVID-19 ISS were compared. There were 3032 cases during the pre-COVID period and 464 cases during COVID-19 period which were included in the analysis. We saw no difference between the median pre-COVID^9^ and COVID-19^9^ ISS (*P* > .05) (Table 1).

**Table 1.** Changes in Trauma Variables Before and After COVID-19.

Topic	Data						
ISS score		Average	Median	Standard deviation	N		*P*-value
	Pre-COVID	10.51	9	9.00	3032		.2018
	COVID	10.18	9	7.23	464		
Injury type		Blunt	Burn	Penetrating	Other	Total	*P*-value
	Pre-COVID	2531 (83.5%)	9 (.3%)	473 (15.6%)	19 (.62%)	3032	.3504
	COVID	409 (84.7%)	1 (.21%)	73 (15.1)	0 (0%)	483	
Blunt vs. MVC		MVC	Other blunt	Total			*P*-value
	Pre-COVID	921 (36.39%)	1610 (63.61%)	2531			.009704
	COVID	119 (29.60%)	282 (70.40%)	402			
Alcohol involvement		Yes	No	Total			*P*-value
	Pre-COVID	475	2545	3020			2.26 × 10^−07^
	COVID	122	358	480			
Average length of stay		Mean	Median	Standard deviation	N		*P*-value
	Pre-COVID	3.87	2	6.04	3033		8.488 × 10^−06^
	COVID	5.39	2	9.58	574		

Abbrevations: ISS, injury severity score; MVC, motor vehicle collision.

Next, the distributions of injury mechanism across the pre-COVID and COVID-19 periods were compared. In the pre-COVID-19 period, there were 2531 blunt traumas (83.5%), 9 burns (.3%), 473 penetrating injuries (15.6%), and 19 other mechanisms of trauma (.62%). In the COVID-19 period, there were 409 blunt traumas (84.7%), 1 burn (.21%), 73 penetrating injuries (15.1%), and 0 other mechanisms of trauma (.0%). Upon analysis, there were no statistically significant differences between the distribution of injury mechanisms pre-COVID and COVID-19 (*P* > .05) (Table 1).

The blunt injuries were further subdivided to compare the distribution of MVC injuries to other blunt injuries. In the pre-COVID-19 period, there were 921 MVC injuries (36.39%) and 1610 other blunt injuries (63.61%). In the COVID-19 period, there were 119 MVC injuries (29.60%) and 283 other blunt injuries (70.40%). There is a statistically significant difference between the 2 distributions (*P* < .05) (Table 1).

The distributions of cases in which alcohol was involved were compared between the 2 periods and there was a statistically significant difference in the distribution of alcohol involvement in the pre-COVID and COVID-19 periods (*P* < .05). In the pre-COVID-19 period, there were 475 cases in which alcohol was involved (15.7%). In the COVID-19 period, there were 122 cases in which alcohol was involved (25.5%). In the pre-COVID-19 period, there were 3033 cases with a documented length of stay and in the COVID-19 period, there were 574 such cases (Table 1).

The mean hospital length of stay in the pre-COVID-19 period (3.87 days) and the mean hospital length of stay in the COVID-19 period (5.39 days) were found to be statistically significant (*P* < .05) (Table 1).

## Discussion

Through this study, we assessed the impact of COVID-19 on trauma presentation patterns at a level 1 academic trauma center. The median ISS was identical before and during the pandemic, indicating a similar distribution of severity per trauma pre-COVID-19 and during COVID-19. This is supported by the trend observed by Rhodes, Petersen, and Biswas in the setting of a rural trauma center who reported that ISS scores did not significantly change after the pandemic.^12^

There was a significant increase in the mean length of stay after COVID-19 but no change in the median length of stay. This finding cannot be fully explained by the data set, but it is possible that some patients have higher rates of complications, either infectious or otherwise following the pandemic. As stated by Grabowski and Mor, fewer nursing homes are accepting short-term postacute care patients.^13^ Therefore, patients may have longer hospital stays during the pandemic due to the lack of options for placement following surgery.

There were no significant changes in distribution of trauma presentations between the 4 major injury mechanisms (blunt, penetrating, burns, and other) during the pre-COVID-19 and COVID-19 periods. However, after subdividing blunt traumas, we see a significant decrease in the proportion of MVC-related traumas. This is consistent with reports that the number of drivers has decreased significantly following COVID-19.^6^ A post hoc analysis to assess differences more specifically may be useful.

After COVID-19, there was a significant increase in the proportion of trauma cases in which alcohol was involved. This might represent an area where psychosocial-related interventions could improve patient outcomes. Overall, more trauma patients are presenting to the hospital intoxicated despite restrictions in locations in which people most typically congregate to consume alcohol.^14^ However, it is possible that patients have been increasingly isolated, which could lead to increased alcohol abuse and dependence.^15^ Interventions should aim to identify these at-risk patients to prevent traumas related to significant alcohol abuse.

Limitations of this study include missing data points from improper charting. This study was conducted using data from a single center, which may not be broadly applicable to different regions with different population dynamics, in particular, in areas with different responses to COVID-19. The study is also retrospective, which limits further investigation into the mechanisms behind the observed changes.

Overall, there has been an expected decrease in number of MVCs during COVID-19, but no change in the remaining distribution of trauma activations. Increased length of stay and increased alcohol-related traumas represent an area where interventions may be of benefit. Future research with a more stratified approach to comparing trauma, such as directly comparing the instances of home injuries to injuries which occur outside of the home, would be useful in elucidating the landscape of trauma during the pandemic. It may also be beneficial to investigate the frequencies of self-harm-associated traumas before and after the pandemic to further analyze COVID-19’s effect on mental health.
